# Injury Risk Factors of the Tennis Serve: A Systematic Review and Meta-Analysis

**DOI:** 10.1177/27536351251374616

**Published:** 2025-10-01

**Authors:** John Bradley, Ben L. Langdown, David Bowmaker, Stewart Kerr

**Affiliations:** 1 Faculty of Wellbeing, Education and Language Studies, School of Education, Childhood, Youth & Sport, The Open University, Milton Keynes, UK; 2 Life Fit Wellness, Falkirk, UK

**Keywords:** sports injury, tennis serve, injury risk, meta-analysis

## Abstract

**Background:**

A key stroke in tennis is the serve. It is the most strenuous stroke in the game and has the greatest potential for injury. The serve has been broken down into a number of key stages to allow analysis of the movements involved.

**Objective:**

The aim of this study was to conduct a systematic review of all injuries related to the stages of the tennis serve and to assess their relative impact on the risk of injury using a meta-analysis.

**Methods:**

A systematic search was conducted across 8 electronic databases, supplemented with searches of grey literature. Eligible studies presented data from injured (injury group) and uninjured (control group) tennis players in a form that allowed further analysis of each risk factor for comparison. All results were presented as effect sizes (Cohen’s *d*) to allow comparison of each risk factor and to identify the most important risk factors associated with injury. Overall study quality was assessed using an adapted Downs and Blacks checklist.

**Results:**

A total of 29 studies were included in the meta-analysis, identifying 130 risk factors. 36 risk factors had an effect size suggesting a large or significant association with injury in tennis players (*d* ⩾ 0.8 or ⩽−0.8). These were divided into general risk factors and those associated with the preparation, acceleration and follow-through phases of the tennis serve. Seven of the risk factors were from 3 or more studies allowing meta-analysis, 3 were from 2 studies and 26 were from a single study. Downs and Black checklist scores ranged from fair to good for the studies included in this review. There was no evidence of publication bias.

**Conclusion:**

Meta-analysis identified dominant shoulder rotation strength, internal rotation range of movement, weekly tennis volume, age, body mass and height as risk factors for injury in the tennis serve.

## Introduction

Tennis is a popular sport around the globe. It is estimated that 87 million, or 1.17% of the world’s population play tennis.^1^ Of this population, in 2019 over 3600 players had a professional ranking, and over 7200 junior players (13-18 years) had an ITF Junior Ranking (i.e., won a match in the main draw of a ranking tournament).^2^ Players become eligible for junior ranking tournaments at 13 years of age, but players can start playing tennis from age 5 to 6 years.^3^ Previous research indicates that considerable training loads can start at a young age, with junior players averaging 9 hours of tennis training, 2 to 3 hours of conditioning training and ⩾2 hours of match play each week.^4-6^ Professional tennis players play ~5 to 6 days a week, with 3 to 4 hours of tennis training and 1 to 2 hours strength and conditioning training each day, depending on which phase of the season they are in.^7-9^ Early specialisation and these increased training loads can eventually lead to players developing an overuse injury.^10^ Overuse injuries represent the most common health problem experienced by tennis players, with 12% of 11 to 14 year old elite junior tennis players experiencing an overuse injury every week.^6^

The most strenuous stroke in tennis is the serve^11,12^ which is associated with the greatest potential for musculoskeletal injury.^13^ The serve plays a key performance role as the dominant stroke in tennis and can account for 45% to 60% of the total strokes played during a service game at senior, professional level,^14^ or an average of 43 serves per set,^15^ with junior players performing slightly less serves in match play than professional players.^15,16^ Given its prevalence within the game, the serve can be considered a suitable focus for injury prevention in tennis.

The tennis serve is a culmination of a proximal-distal movement sequence through a kinetic chain, starting with the lower limbs generating ground reaction forces and continuing via the trunk and finishing in the upper limb moving the racket through impact with the ball.^17,18^ Any of these movements involved in the kinetic chain may present a potential injury risk. Indeed, several studies have identified a number of injury risk factors associated with the serve in tennis (e.g.^4,19-21^). The serve has previously been broken down into a number of stages to identify the key movements involved and this can be used to identify potential areas of injury (e.g.^17,22-25^). The aim of this study was to conduct a systematic review and meta-analysis of all injuries associated with the serve in tennis and identify those related to the stages of the tennis serve. These resulting risk factors were then aligned t o the 8-stage model of the tennis serve^22^ to assess their relative impact on the risk of injury through a meta-analysis.

## Methods

A systematic search of the available literature was performed in 2022. A total of 8 databases were searched from inception to 2021 (PubMed [Open Access], MEDLINE [EBSCOhost], SPORTDiscus [EBSCOhost], Academic Search Complete [EBSCOhost], AMED [EBSCOhost], CINAHL [EBSCOhost], Web of Science [Clarivate Analytics], and ScienceDirect [Open Access]). Grey literature was also searched for in Google and Google Scholar (https://scholar.google.co.uk/). These were supplemented by cross-referencing to publications cited by authors from the initial literature search. The searches were re-run (April 2024) prior to the final analysis to identify and retrieve any further suitable studies.

The search terms used, initial results and example search strategy are listed in Appendix 1. This systematic review was conducted in line with the recommendations of the Preferred Reporting Items for Systematic Review and Meta-Analysis (PRISMA) guidelines.^26^ The study was registered in the PROSPERO international database of systematic reviews prior to study initiation (registration number: CRD42021277344).

### Study Selection

The literature search included all prospective cohort studies, retrospective case-control studies and cross-sectional studies presenting data from injured (injury group) and uninjured (control group) tennis players in a form that allowed further meta-analysis from each risk factor for comparison. Studies were excluded if the data did not relate solely to tennis, was presented in a form not allowing further meta-analysis, or the main sport was not tennis. Following the search, results were imported into a reference manager, where duplicates were removed, and an initial screening of titles and abstracts was performed by 1 researcher and checked for consistency and completeness by other members of the authorship. Any disagreements were resolved using the following inclusion/exclusion criteria as a basis for discussion (Table 1).

**Table 1.** Inclusion and Exclusion Criteria.

Inclusion criteria	Exclusion criteria
Studies were included if they meet all of the following criteria:	Studies were excluded if they meet any of the following criteria:
Primary data is presented or analysis of existing data sets	No data or new analysis of existing data sets is presented
Is an original investigation	Is a review
Data presented in two groups: injured and uninjured tennis players	Is a case study
Relates to tennis and tennis players	Does not relate to the sport of tennis
Includes human participants (these can be para-athletes	Solely uses computer modelling

**Figure 1.**
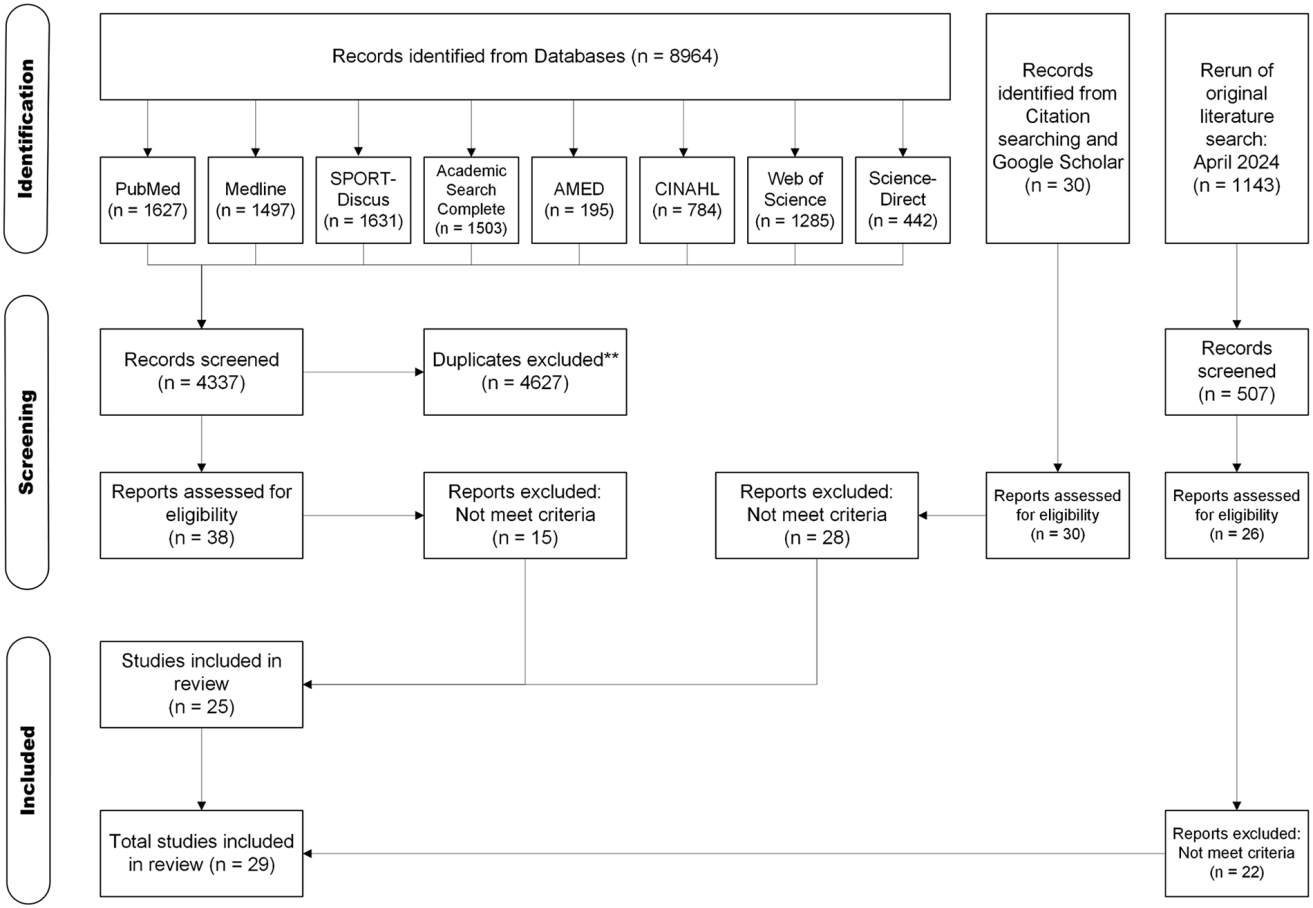
PRISMA flow diagram showing the identification and selection of suitable studies, based on PRISMA guidelines.^26^ **Following initial screening.

Where records failed to meet the inclusion criteria, they were excluded from any further analysis. An inclusive approach was taken to the initial screen and where uncertainty remained, the record was included for further assessment of the full text against inclusion criteria. Studies written in a foreign language were translated into English (n = 2) and screened for eligibility. At least 2 attempts were made, a month apart, to contact original authors to locate publicly unavailable full texts, obtain missing data or clarify data analysis. Where these records were unavailable the results were not inferred, and the records were omitted from the review. The full PRISMA flowchart that documents the process outlined above can be found in Figure 1.

### Statistics and Data Analysis

To allow comparison, all the results are presented as effect sizes (Cohen’s *d*) to measure the magnitude of effect of each risk factor and allow comparison of the practical significance of all the different studies, independent of sample size. The results were converted from odds ratio using the methods of Chinn^27^ and Borenstein,^28^ or from means and standard deviation or F-test using the method of Thalheimer and Cook.^29^ Converting all measures of effect size to the Cohen’s *d* effect size assumes that these studies are all comparable. Even if these assumptions do not hold exactly, omission of any studies using different forms of effect size would result in a potential loss of information and reduce the depth of the review.^28^ To facilitate comparison between different studies, the convention described by Cohen^30^ was adopted of Small Effect Size: *d* ⩾ 0.2; Medium Effect Size: *d* ⩾ 0.5; Large Effect Size: *d* ⩾ 0.8.

Each risk factor was aligned to a stage of the serve based on the descriptions outlined by Kovacs and Ellenbecker,^22^ supplemented by the knowledge and experience of the authors. Any risk factors not aligning to a stage of the tennis serve but coming from studies that were investigating injuries in the tennis serve were assigned as general risk factors.

The inclusive nature of this literature review resulted in many risk factors for injuries in the tennis serve being based on 1 or 2 studies. When 3 or more studies investigated the same risk factor, the overall effect on tennis injuries was assessed using Comprehensive Meta-Analysis software (version 4)^31^ and MetaAnalysisOnline.com.^32^ A random effects model was used for a simple meta-analysis, testing if the mean effect size of any of the risk factors were significantly associated with injuries in the tennis serve. Of the 130 risk factors investigated, only 16 were from 3 or more studies. The majority of these were General risk factors (Figure 3). The results of the meta-analysis is shown in Table 3.

**Figure 2.**
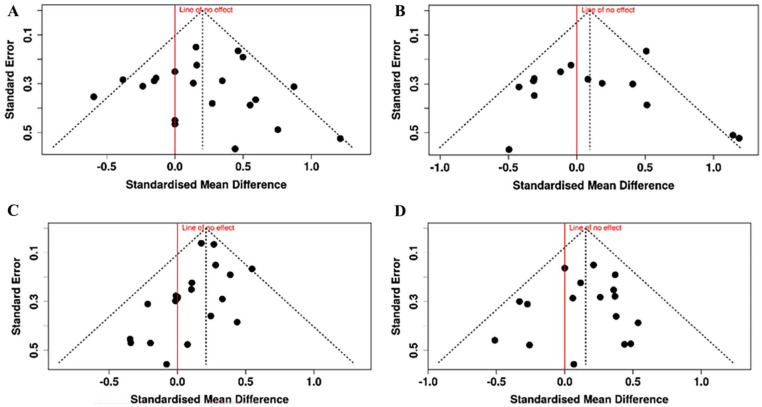
Funnel plots^32^ of Cohen’s *d* effect size against standard error for the risk factors calculated from more than 10 studies. Red line shows line of no effect. Egger’s linear regression test does not support the presence of funnel plot asymmetry or publication bias in any of the plots. (A) Age as risk factor for injury; (B) years playing tennis as risk factor for injury; (C) body mass as risk factor for injury; (D) height as risk factor for injury.

Studies selected as suitable for inclusion in this review were assessed for study quality and any risk of bias, using an adapted Downs and Black checklist.^33^ This was presented as a score out of 15 and assigned a corresponding quality level adapted from Hooper et al^34^ (Table 2; full analysis listed in Appendix 2). When the risk factor was from 2 or more studies, the quality levels were mapped according to the strength of their evidence.^34^ The evidence levels are as follows: 1a (very strong): the findings are supported by the results of 2 or more studies of at least excellent quality; 1b (strong): the findings are supported by at least 1 study of excellent quality; 2a (moderately strong): the findings are supported by 2 or more studies of at least good quality; 2b (limited): the findings are supported by at least 1 study of good quality; 2c (weak): the findings are supported by at least 1 study of fair or poor quality; 3 (consensus): in the absence of evidence, there is agreement by a group of experts on the appropriate treatment course; and 4 (conflicting): there is disagreement between the findings of at least 2 randomised controlled trials. Funnel plots were used to assess any risk of publication bias (Figure 2). The nature of the studies included in this review gave the possibility that several studies may use the same participants in their analysis to compare the same variable. Where it was possible to identify those studies, data from 1 of the studies was excluded to avoid duplication.

**Table 2.** Participant Demographics and Characteristics of Studies Included in this Review.

Author(s)	Study type; (evidence level*)	Participant demographics: n, age (SD), gender split	Country	Participant tennis level	Data collected	Method of pain reporting	Time loss or non-time loss injury
Campbell et al^35^	Case-control (Good)	Participants: 20 Age: 16.0 (1.21) y 20 M; 0 F	Australia	National Academy Institute of Sport	Serve: Lumbar kinetics & kinematics	Self-reported survey of LBP 15 mo before/after data collection, confirmed by physician	Time-loss injury
Campbell et al^36^	Case-control (Good)	Participants: 20 Age: 16.0 (1.21) y 20 M; 0 F	Australia	National Academy Institute of Sport	Serve: Lumbar kinetics & kinematics	Self-reported survey of LBP 15 mo before/after data collection, confirmed by physician	Time-loss injury
Campbell et al^37^	Case-control (Good)	Participants: 19 Age: 16.2 (1.0) y 19 M; 0 F	Australia	National Academy Institute of Sport	Groundstroke: Lumbar kinetics & kinematics	Self-reported survey of LBP 15 mo before/after data collection, confirmed by physician	Time-loss injury
Dakic et al^38^	Prospective cohort (Good)	Participants: 52 Age: 24.8 (4.85) y 0 M; 52 F	Grand Slam tournament	WTA Professional	General injuries: WTA medical records; self-reported training volume)	Tennis-related injury recorded on WTA medical documentation system	Non-time-loss injury
Gillet et al^5^	Case-control (Good)	Participants: 91 Age: 8-15 y 91 M; 0 F	France	Regional tennis centre of excellence	Shoulder strength and RoM	Self-reported survey of shoulder problems in last 12 mo, confirmed by health record	Non-time-loss injury “Injury affecting tennis play”
Gillet et al^4^	Case-control (Good)	Participants: 28 Age: 12.1 (2.5) y 28 M; 0 F	France	International tennis number: 3-6	Serve: upper body kinematics	Self-reported survey of shoulder problems in last 12 mo, confirmed by health record	Non-time-loss injury “Injury affecting tennis play”
Grosdent et al^39^	Case-control (Good)	Participants: 38 Age: 25.3 (5.6) y 38 M; 0 F	Belgium	Top 150 ranked players in Belgium	Trunk muscle strength and RoM	Self-reported questionnaire for current LBP, confirmed by physiotherapist	Non-time-loss injury
Grosdent et al^40^	Case-control (Good)	Participants: 35 Age: 17.4 (2.6) y 9 M; 26 F	Belgium	ATP, WTA, ITF ranked players	Questionnaire and lumbopelvic mobility	Questionnaire	Time-loss injury
Hang and Peng^41^	Cross-sectional survey (Fair)	Participants: 534 Age: 14-63 y 428 M; 106 F	Taiwan	Intermediate to highly skilled tournament players	Questionnaire: Injury location and tennis experience.	Interview and examination for tennis-related upper body trauma	Time-loss injury
Hjelm et al^20^	Prospective Cohort (Good)	Participants: 55 Age: 15.4 (2.9) y 35 M; 20 F	Sweden	Club players	Questionnaire; Muscle strength and RoM	Questionnaire and investigator follow-up.	Non-time loss injury (“injury preventing playing at 100%”)
Jörger and Leonhard^42^	Retrospective cohort (Fair)	Participants: 117 Age: 35-82 y 82 M; 35 F	Germany	Regional and National tournament players	Questionnaire: fitness and injuries.	Questionnaire	Non-time-loss injury
Johansson et al^43^	Longitudinal cohort (Good)	Participants: 301 Age: 14.5 (2.0) y 176 M; 125 F	Sweden	Swedish Tennis High Performance programme	Questionnaire: SMASH cohort study	Questionnaire	Non-time loss injury
Johansson et al^44^	Longitudinal cohort (Good)	Participants: 271 Age: 14.5 (2.0) y 56 M; 215 F	Sweden	Swedish Tennis High Performance programme	Questionnaire: SMASH cohort study	Questionnaire	Non-time loss injury
Kim et al^45^	Case-control study (Good)	Participants: 13 Age: 21.5 (1.0) y ? M; ? F	Korea	Korean professional players	Serve: Upper body kinematics	Positive shoulder impingement tests	-
Levy et al^46^	Case-control (Good)	Participants: 28 Age: 20.5 (3.6) y ? M; ? F	Australia	Internationally ranked players	MRI of wrist	Self-reported wrist pain	Non-time-loss injury
Lucado et al^47^	Case control (Good)	Participants: 42 Age: 45.9 (7.7) y 0 M; 42 F	USA	Recreational, non-professional players	Upper body strength and RoM	Screening tests for elbow pain	Non-time-loss injury
Marcondes et al^48^	Case-control (Good)	Participants: 49 Age: 26.2 (3.8) y 49 M; 0 F	Brazil	Competitive amateur players	Shoulder RoM, strength and tightness	Positive shoulder pain following tennis match and in screening tests for shoulder pain	Non-time-loss injury
Martin et al^49^	Prospective cohort (Good)	Participants: 20 Age: 24.7 (5.6) y 20 M; 0 F	France	Professional and national tennis number from 4 to 1.	Upper body kinetics	Questionnaire: injuries in 2 seasons after data collection, confirmed by coach or physiotherapist	Non-time-loss injury
Martin et al^24^	Prospective cohort (Good)	Participants: 19 Age: 25.1 (5.9) y 19 M; 0 F	France	International tennis number from 4 to 1.	Energy flow, injury questionnaire	Questionnaire: injuries in 2 seasons after data collection, confirmed by coach or physiotherapist	Non-time loss injury (“injury preventing playing at 100%”)
Moreno-Pérez et al^50^	Case-control (Good)	Participants: 47 Age: 23.2 (4.9) y 47 M; 0 F	ATP World Tour	World ranked top 1000	Shoulder rotation RoM	Self-reported history of disabling shoulder pain	Time loss injury
Moreno-Pérez et al^51^	Case-control (Good)	Participants: 61 Age: 20.2 (5.1) y 61 M; 0 F		Members of ATP or ITF World Tour	Groyne injuries and hip strength	Questionnaire reporting history of disabling groyne pain	Time loss injury
Moreno-Pérez et al^52^	Case-control (Good)	Participants: 58 Age: 20.7 (4.9) y 58 M; 0 F	Spain	Spanish academy members	Shoulder strength and RoM	MRI or ultrasound confirmed shoulder pain	Non-time loss injury
Moreno-Pérez et al^53^	Case-control (Good)	Participants: 64 Age: 19.6 (3.0) y 42 M; 22 F		ATP/WTA Ranking: 726.6 ± 562	Hip RoM	Self-reported LBP	Time loss injury
Nigg et al^54^	Prospective cohort (Good)	Participants: 171 Age: 25.8 (8.8) y 132 M; 39 F	Canada	Club or University players	Injury status, shoe type	Physician-completed medical questionnaire	Non-time loss injury
Priest et al^55^	Case-control (Fair)	Participants: 2516 Age: 9-69 y 1277 M; 1239 F	USA	California tennis school	Anthropometric variables and injury history	Self-reported questionnaire on elbow pain, confirmed by examination	Non-time loss injury
Queiroz et al^56^	Cross-sectional (Fair)	Participants: 159 Age: 45.3 (11.4) y 142 M; 17 F	Brazil	Rio de Janeiro tennis clubs	Injury history and training characteristics.	Structured questionnaire and interview	Non-time loss injury
Rogowski et al^57^	Case-control (Good)	Participants: 147 Age: 11-30 y ? M; ? F	France	International tennis number: from 10 to 1.	Anthropometric variables and injury history	Supervised questionnaire on history of upper limb injuries	Time loss and non-time-loss injury
Stanley et al^58^	Case-control (Good)	Participants: 51 Age: 44.9 (13.0) y 0 M; 51 F	Australia	Amateur tennis league players	Shoulder strength and RoM	Questionnaire on history of shoulder pain	Non-time loss injury
Vad et al^59^	Case-control (Fair)	Participants: 100 Age: 25.4 (17-37) y 100 M; 0 F	ATP Circuit	Professional ATP circuit players	Hip and Shoulder RoM	Self-reported LBP	Non-time loss injury (“Limiting playing”)

Abbreviation: RoM, range of motion.

* Evidence Level is adapted from Downs and Black analysis,^33^ and assigned a quality level.^34^

## Results

### Search Results

The initial database searches yielded a total of 8964 results, with 4627 duplicates removed in the reference manager. The remaining 4337 results were reviewed at title and abstract only level with 38 retained for a full paper retrieval and review. Following further searches on Google and Google Scholar and based on reference lists, a further 30 were included. This made a total of 68 for review, and following retrieval and review, 25 were included in the present manuscript. A rerun of the original search in April 2024 yielded 1143 results, with 636 duplicates removed in the reference manager. Five hundred and seven were reviewed at title and abstract level with 26 retained for a full paper retrieval and review. This led to a further 4 papers being included in the present manuscript. The full PRISMA flowchart can be found in Figure 1.

Demographic variables of the included studies are presented in Table 2. A total of 5126 participants were included (3055 males; 2041 females with 2 studies unspecified), with a mean *n* of 177 ± 463 participants per study (median = 52 participants; range: 13-2516 participants). Of the 29 studies selected for analysis, 19 were case-control studies, 5 were prospective cohort studies, 1 was a retrospective cohort study, 2 were longitudinal cohort studies, and 2 were cross-section studies, 11 studies included both male and female tennis players, 13 used only male players, 3 used only female players and 2 studies did not specify the gender of their participants.

### Risk Factors

From our results, 130 risk factors were found to be associated with injury in the tennis serve, of which 30 risk factors had an effect size suggesting a large association with injury in tennis players (results ⩾ 0.8 or ⩽−0.8). Of these 130 risk factors, 16 were from 3 or more studies. To see if these risk factors were significantly associated with tennis injuries, a random-effects meta-analysis was carried out (Table 3) with forest plots of the meta-analysis results presented in Appendix 3. This showed a further 6 risk factors were significantly (*P*<.05) associated with injury in tennis players. The results illustrate general injury risk factors and injury risk factors associated with each of the 3 serve phases from the 8-stage model of the tennis serve from Kovacs and Ellenbecker^22^ (preparation, acceleration and follow-through).

**Table 3.** Results of the Random-Effects Meta-Analysis for All Risk Factors from Multiple Studies.

Amend table risk factor	Stage	Number of studies	Mean effect size	95% confidence interval	*Z*-value	*p*-value
Body mass	General risk factors	20	0.209	0.138-0.281	5.730	<.001
Age	General risk factors	21	0.202	0.043-0.362	2.480	.013
Height	General risk factors	17	0.153	0.027-0.280	2.380	.017
Years playing tennis	General risk factors	14	0.095	−0.127 to 0.318	0.840	.401
Weekly tennis	General risk factors	9	0.159	0.009-0.305	2.080	.037
Training volume	General risk factors	7	0.099	−0.068 to 0.265	1.160	.245
Gender	General risk factors	5	0.073	−0.100 to 0.246	0.824	.410
Serve speed/racket velocity	General risk factors	4	−0.106	−0.546 to 0.335	−0.470	.638
Dominant ER/IR strength ratio	Stage 4: Cocking phase	4	−0.608	−0.941-−0.275	−3.579	<.001
Dominant shoulder ER strength	Stage 4: Cocking phase	4	−0.384	−0.720-−0.048	−2.238	.025
Non-dominant shoulder TAM	Stage 4: Cocking phase	4	−0.402	−1.033 to 0.230	−1.246	.213
Non-dominant shoulder ER RoM	Stage 4: Cocking phase	6	−0.177	−0.466 to 0.111	−1.204	.228
Dominant shoulder TAM	Stage 4: Cocking phase	4	−0.326	−0.974 to 0.322	−0.985	.324
Dominant shoulder ER RoM	Stage 4: Cocking phase	6	0.144	−0.392 to 0.680	0.527	.598
Dominant shoulder IR strength	Stage 6: Contact	4	0.117	−0.165 to 0.399	0.815	.415
Dominant shoulder IR RoM	Stage 7: Deceleration	7	−1.118	−2.362 to 0.126	−1.760	.078

Abbreviations: ER, external rotation; IR, internal rotation; RoM, range of motion; TAM, total arc of motion.

### Preparation Phase: Stage 1: Start and General Risk Factors

**Figure 3.**
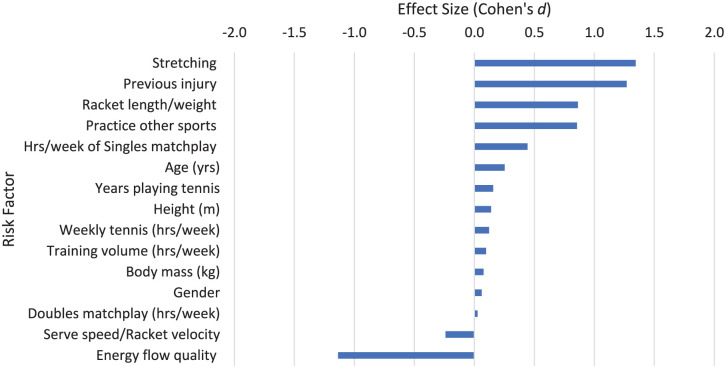
General risk factors and risk factors relating to stage 1 of the tennis serve.

No specific risk factors based on the movements involved in stage 1 were found. General risk factors relating to tennis (Figure 3) included increased static stretching before and after tennis play (*d* = 1.345), sustaining a previous injury (*d* = 1.270), practicing other sports (*d* = 0.855) and increased tennis racket length/weight (*d* = 0.864) which all have a large association with tennis injury. The risk factor for the impact of sustaining a previous injury on risk of tennis injury (*d* = 1.270) suggested a significant association with injuries in tennis. Energy flow is defined as the movement of energy in the human body as the tennis serve takes place - this includes the generation and absorption of energy at the joints and transfer of mechanical energy between segments in the kinetic chain.^24^ Greater energy flow quality (more effective transfer of mechanical energy through the kinetic chain, from the trunk to the arm and hand) shows a large association with reduced injury risk (*d* = −1.137). The meta-analysis also suggested that despite low Cohen’s *d* effect sizes from the participant characteristics of age (*d* = 0.202), body mass (*d* = 0.209), height (*d* = 0.153) and weekly tennis volume (*d* = 0.159), the large number of studies reporting these factors suggested that an increase in age, body mass, height and weekly tennis volume were all significantly associated with an increase in tennis injuries (*P* = .013; *P* < .001; *P* = .017; *P* = .037 respectively). All other risk factors have a medium effect size or less.

### Preparation Phase: Stage 2: Release

**Figure 4.**
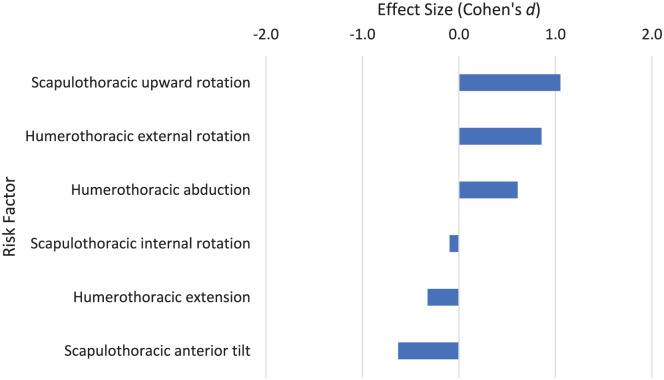
Risk factors relating to stage 2 of the tennis serve: Ball release.

In stage 2 of the serve from the start up to the point of ball release (Figure 4), increased scapulothoracic upward rotation (*d* = 1.052) and increased humerothoracic external rotation *d* = 0.856) in the dominant shoulder show a large association with a history of shoulder injury.^4^ An increase in humerothoracic abduction of the shoulder at ball release shows a moderate association with injury (*d* = 0.609). This suggests the ball toss in the preparation phase may have an implication on injury in the serving arm of tennis players. This may in turn link to the ball position at the point of impact and influence risk factors at this stage. An increase in humerothoracic extension (*d* = −0.325) and scapulothoracic anterior tilt at ball release (*d* = −0.48) showed a moderate association with a reduction in injury.

### Preparation Phase: Stage 3: Loading

**Figure 5.**
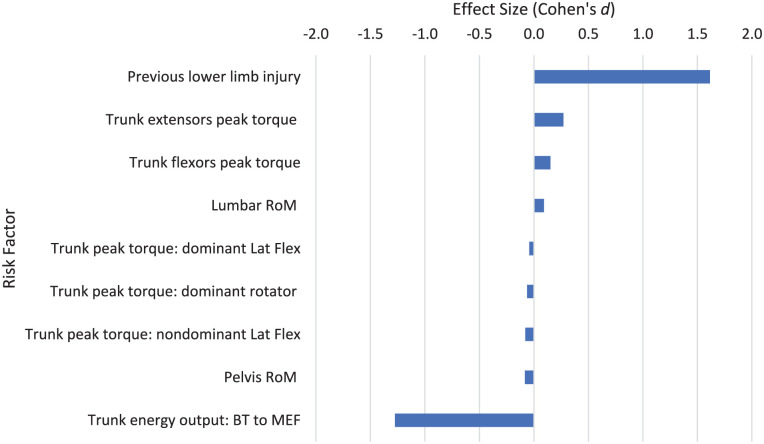
Risk factors relating to stage 3 of the tennis serve: loading. Abbreviations: BT, ball toss; MEF, maximal elbow flexion; RoM, range of motion; Lat Flex, lateral flexion.

The mechanics of the loading stage of the tennis serve (Stage 3) can influence subsequent stages of the serve. The risk from a previous lower limb injury was included in this section (*d* = 1.615) as optimal leg drive can influence whole body movement and reduce shoulder load during the serve.^12^ Perhaps linked to serve mechanics and optimal leg drive, increased energy output from the trunk (generated initially by the ground reaction forces from the leg drive) to the upper limbs has a large association with reduction in injury (*d* = −1.275; Figure 5). To achieve the optimum position ready for the subsequent cocking phase, lumbar flexibility is suggested to be important,^22^ however, the effect sizes suggest Lumbar RoM here only has a small impact (*d* = 0.092) on injury risk.

### Preparation Phase: Stage 4: Cocking

**Figure 6.**
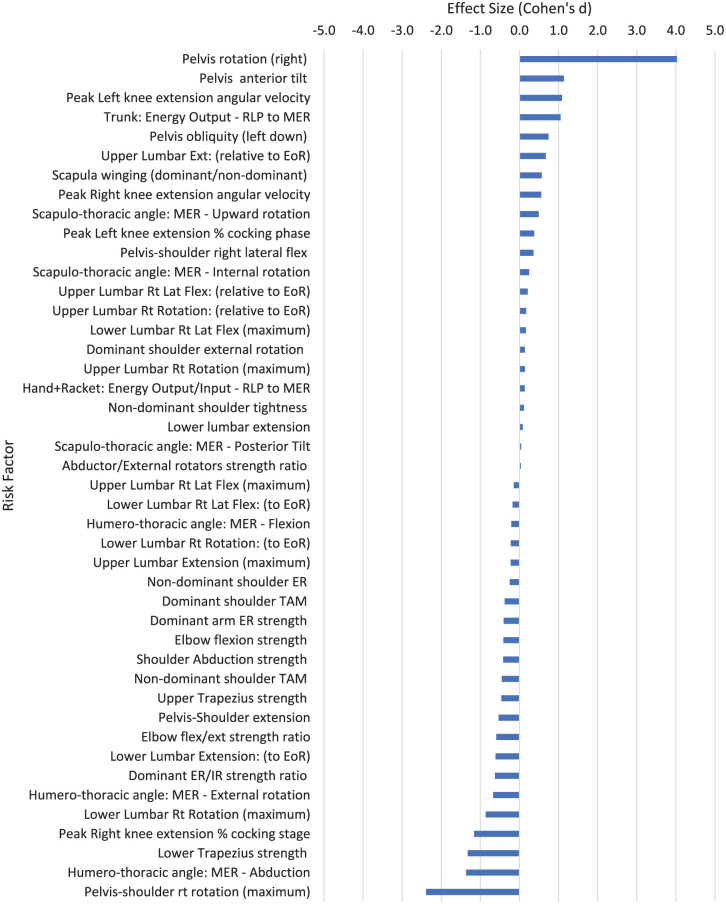
Risk factors relating to stage 4 of the tennis serve: cocking. Abbreviations: EoR, end of range; ER, external rotation; IR, internal rotation; Lat Flex, lateral flexion; MER, maximal external rotation of the shoulder; RLP, racket low point; Rt, right; TAM, total arc of motion (sum of IR and ER range of motion).

The cocking stage (Stage 4) and the subsequent acceleration stage are the most dynamic phases of the tennis serve involving the greatest muscle activation.^60^ In stage 4, the racket arm is “cocked,” or externally rotated and positioned ready for the acceleration phase (stage 5) to follow. The legs simultaneously extend and drive the body upwards to increase serve velocity, known as the drive phase or late cocking phase in some publications (e.g.^24,36^). The risk factors with a large association with injury in the cocking stage (*d* ⩾ 0.8) are increased pelvis rotation (right, or away from the target; *d* = 4.030), increased anterior pelvic tilt (*d* = 1.138) and greater peak angular velocity for left knee extension (lead knee in the right handed player; *d* = 1.090; Figure 6). Those risk factors having a large association with reduced injury risk (*d* ⩽ −0.8) are a later timing of peak right knee extension in the cocking stage (*d* = −1.163), greater lower trapezius strength (*d* = −1.323) and greater pelvis-shoulder rotation away from the target (*d* = −2.387). Meta-analysis also showed that the increased dominant shoulder ER strength and increased dominant arm ER/IR strength ratio both had a significant association with reducing injuries in the tennis serve (*P* = .025; *P* < .001 respectively; Table 3).

### Acceleration Phase: Stage 5: Acceleration

**Figure 7.**
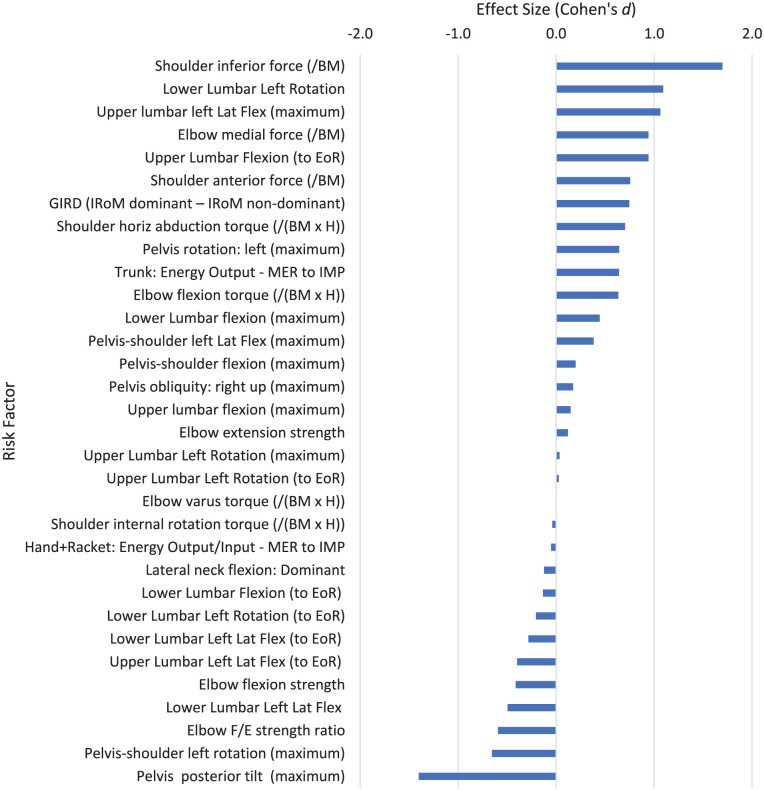
Risk factors relating to stage 5 of the tennis serve: acceleration. Abbreviations: BM, body mass; EoR, end of range; F/E: flexion/extension; GIRD, glenohumeral internal rotation deficit; IMP, ball impact; H, height; IRoM, internal range of motion; Lat Flex, lateral flexion; MER, maximal external rotation of the shoulder.

The acceleration phase of the tennis serve (Stage 5) is closely linked to the cocking/drive phase and culminates in the impact of the tennis ball with the racket. This is represented by the forward swing phase in some publications. From inverse dynamics analysis of the tennis serve, 2 strength measures were strongly associated with risk of injury: high shoulder inferior force, or downwards movement of the head of the humerus (*d* = 1.697) and high elbow medial force (*d* = 0.943; Figure 7). Conventional dynamometer strength testing showed a high elbow flexion/extension strength ratio had a moderate association with a reduced risk of injury (*d* = −0.593; i.e., greater elbow flexion strength reduces the risk of injury). Lumbar kinematics are strongly linked to lower back pain (LBP). Greater lower lumbar left rotation (*d* = 1.093; i.e., greater rotation towards the target) and upper lumbar left lateral flexion (*d* = 1.064; i.e., greater lateral flexion of the lead side) are associated with greater risk of LBP injury. Greater pelvis posterior tilt (*d* = −1.402) is strongly associated with a reduction in LBP injuries.

### Acceleration Phase: Stage 6: Contact

**Figure 8.**
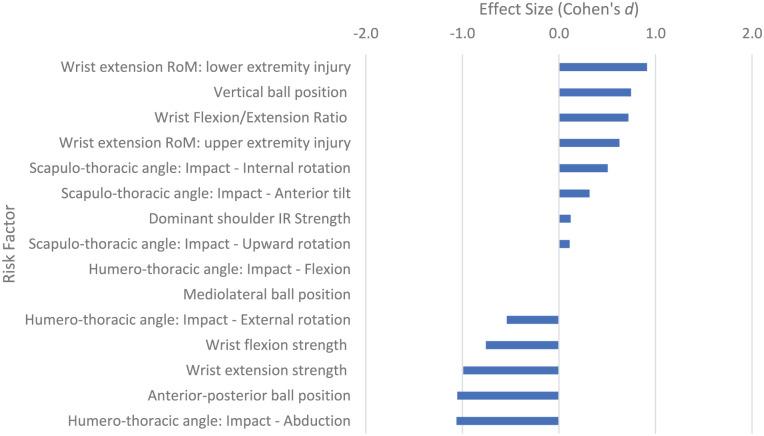
Risk factors relating to stage 6 of the tennis serve: contact. Abbreviations: IR, internal rotation; RoM, range of motion.

At the end of the acceleration phase at the point of ball contact the wrist can play a key role in final racket head velocity.^61^ Wrist extension RoM has a strong association with lower limb injury (*d* = 0.911) and moderate association with upper limb injury (*d* = 0.625). The wrist extension strength has a strong association with a reduction in lateral epicondylalgia injuries (*d* = −0.991) and the wrist flexion strength is also associated with a moderate reduction in risk of these injuries (*d* = −0.757). This perhaps suggests wrist extension strength is more important as the wrist flexion/extension strength ratio also has a moderate association with increased injury risk (*d* = 0.718). A higher vertical position of the ball at the point of impact is strongly associated with lower back pain (*d* = 0.745) whereas a more anterior position of the ball at impact is strongly associated with a reduction in injury risk (*d* = −1.054, Figure 8). A greater humero-thoracic abduction angle had a strong association with reduced risk in the tennis serve (*d* = −1.061) and a greater humero-thoracic external rotation angle at ball impact had a moderate association with reduced injury risk (*d* = −0.542). Greater scapula-thoracic internal (*d* = 0.569) and upward rotation (*d* = 0.519) angles had a moderate association with risk of injury.

### Follow-Through Phase: Stage 7: Deceleration

**Figure 9.**
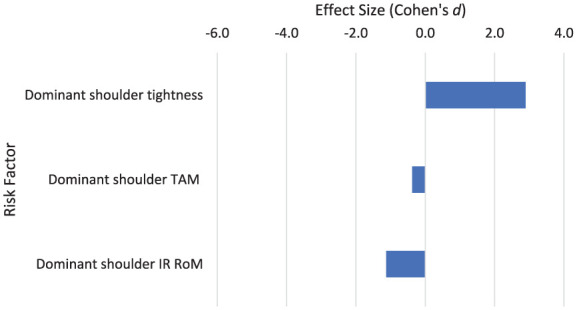
Risk factors relating to stage 7 of the tennis serve: deceleration. Abbreviations: IR, internal rotation; RoM, range of motion; TAM, total arc of motion.

In stage 7 of the tennis serve, when the racket is decelerating after impact, increased posterior dominant shoulder tightness (*d* = 2.894) has a large association with increased risk of injury (Figure 9). Consistent with this, an increased dominant shoulder IR RoM has a large association with a reduction in injury (*d* = −1.118; *P* = .078; Table 3).

### Follow-Through Phase: Stage 8: Finish

**Figure 10.**
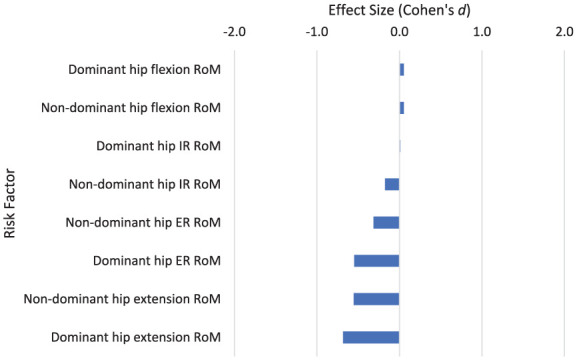
Risk factors relating to stage 8 of the tennis serve: Finish. Abbreviations: ER, external rotation; IR, internal rotation; RoM, range of motion.

The lower limbs are important for providing deceleration following ball contact in the tennis serve. Players typically land on the non-dominant leg following a tennis serve and a greater non-dominant hip extension RoM is associated with a moderate reduction in LBP injury risk (*d* = −0.554; Figure 10). Greater dominant leg hip extension RoM is also associated with a moderate reduction in injury risk (*d* = −0.683), perhaps contributing to the balance on landing.

## Discussion

This review aimed to identify and evaluate the risk factors associated with injury in each of the modelled phases^22^ of a flat tennis serve. Results indicated that of the 130 risk factors that had an association with injury in the tennis serve, 36 risk factors had an effect size suggesting a large association with injury (results ⩾ 0.8 or ⩽−0.8) or were significantly associated with injury following meta-analysis. For ease of analysis, this discussion will split into general risk factors and those that relate to the 3 phases of Kovacs and Ellenbecker’s 8-stage model.^22^ Each phase will be discussed in turn, and can then be used to inform athlete assessment, monitoring and strength and conditioning interventions to protect against these known risks.

### Quality of Evidence

The inclusive nature of this review resulted in some heterogeneity among the included studies. By converting the results into Cohen’s *d*, we were able to compare results from different studies. However, converting between different measures makes assumptions about the data and underlying effects. For example, using Cohen’s *d* can potentially give a biased estimate for the effect size from studies with a small sample size (n < 20).^62^ Of the 29 studies included in this review, 3 studies had less than 20 participants. While this may have added a small risk of bias to the results, the intuitive interpretation of Cohen’s *d* effect sizes was felt more suitable for the wide-ranging remit of this literature review. Publication bias can also be a feature of systematic reviews. Funnel plots of the risk factors composed of greater than 10 studies suggested there was no publication bias in the studies used in this review (Figure 2).

The definition of an injury varied across the studies and may account for some of the variation in the findings. Some of the studies (7/25, 28%; Table 2) used inability to practice or compete as a primary indicator of injury (commonly known as a “time-loss” injury), sometimes confirmed by physician examination. The majority (17/25, 68%) defined injury as requiring medical assistance, or tissue injury confirmed by medical assessment, or injuries that do not result in loss of time from practice or competition (“non-time loss injury”) and managed by physiotherapists. One study (4%) recorded both “time-loss” and “non-time-loss” injuries. The higher “non-time-loss” injuries suggests players continue to play despite injury problems, and agrees with the observation that 1 in 8 players routinely play with pain.^6^ Three studies were a retrospective history of injury,^4,5,42^ while 4 studies reported prospective injury within a group of players.^20,38,49,54^ The participants recruited also varied, from junior players (for example^20^), to amateur adult male and female players (for example^48,58^), to elite senior male and female players (for example^38,52^). Skill level can also influence injury risk. Professional level players have been shown to be less susceptible to upper limb injuries than national or regional level players, suggested to be due to the higher level players having developed a better energy transfer and more efficient serve technique, being able to maximise ball velocity with lower joint kinetics.^23^

There were 26 risk factors with a large Cohen’s *d* effect size resulting from only 1 study. All these studies had a Downs and Black quality level of Good (Table 4). However risk factors from a single study will not carry as much weight of evidence as risk factors resulting from 2 or more studies. Those from 2 or more studies allowed the calculation of an evidence level and 95% confidence interval to confer greater weight of evidence and those from 3 or more studies allowed a meta-analysis to be carried out. The 10 risk factors from 2 or more studies had an evidence level of moderately strong or limited (Table 4). The results from the meta-analysis of the 7 risk factors with a large effect size showed that 4 had a moderate strength of evidence and 3 had a low strength of evidence, based on the size of the confidence interval. This discussion will confer greater evidence weight to risk factors from 2 or more studies, and use risk factors from a single study to support the discussion as necessary.

**Table 4.** Summary Table of All Risk Factors with a Large Cohen’s *d* Effect Size or a Significant Effect Following Meta-Analysis, With Study Characteristics and Quality or Evidence Level.

Phase of tennis serve	Risk factor	Mean ES (Cohen’s *d*)	Results of meta-analysis where appropriate (95% CI, *P*-value, *z*-value)	Reference; total participants (M: male; F: female) x̅: mean age (y)	Downs and Black quality level (1 study) or evidence level (>1 study)
General risk factors	Body mass	0.209	CI: 0.138-0.281; *P* < .001; *z* = 5.730	Refs^4,5,24,34,35,37,41-43,45-51,53,54,56^; N = 3867 (2143M, 1711F); *x̅* = 22.3	Moderately strong (19 studies)
	Age	0.202	CI: 0.043-0.362; *P* = .013; *z* = 2.480	Refs^4,5,20,24,34,35,37,38,41-51,54,56^; N = 1469 (910M, 518F); *x̅* = 21.8	Moderately strong (21 studies)
	Height	0.153	CI: 0.027-0.280; *P* = .017; *z* = 2.380	Refs^4,5,24,34,35,37,41-43,45,47-51,54,56^; N = 1302 (817M, 472F); *x̅* = 22.1	Moderately strong (17 studies)
	Weekly tennis	0.159	CI: 0.009-0.305; *P* = .037; *z* = 2.080	Refs^4,5,20,40,43,44,47,53,57^; N = 1034 (437M, 450F); *x̅* = 16.1	Moderately strong (9 studies)
	Racket length/weight	0.864	CI: 0.063-1.664	Refs^20,41^; N = 55 (35M, 20F); *x̅* = 15.4	Limited (2 studies)
	Stretching	1.345		Ref^20^; N = 55 (35M, 20F); *x̅* = 15.4	Good (1 study)
	General previous injury	1.270		Ref^20^; N = 55 (35M, 20F); *x̅* = 15.4	Good (1 study)
	Practice other sports	0.855		Ref^20^; N = 55 (35M, 20F); *x̅* = 15.4	Good (1 study)
	Energy flow quality	−1.137		Ref^24^; N = 19 (19M, 0F); *x̅* = 25.1	Good (1 study)
Preparation phase: ball release	Scapulothoracic upward rotation	1.052	CI: 1.030-1.074	Refs^4,45^; N = 41 (28M, 0F); *x̅* = 15.1	Moderately strong (2 studies)
	Humerothoracic external rotation	0.856		Refs^4,44^; N = 28 (28M, 0F); *x̅* = 12.1	Good (1 study)
Preparation phase: Loading	Previous lower limb injury	1.615		Ref^20^; N = 55 (35M, 20F); *x̅* = 15.4	Good (1 study)
	Trunk energy output: BT to MEF	−1.275		Ref^24^; N = 19 (19M, 0F); *x̅* = 25.1	Good (1 study)
Preparation phase: Cocking	Dominant shoulder ER strength	−0.384	CI: −0.720-−0.048; *P* = .025; *z* = −2.238	Refs^47,48,52,58^; N = 200 (107M, 93F); *x̅* = 33.5	Moderately strong (4 studies)
	Dominant shoulder ER/IR strength ratio	−0.608	CI: −0.941-−0.275; *P* < .001; *z* = −3.579	Refs^47,48,52,58^; N = 200 (107M, 93F); *x̅* = 33.5	Moderately strong (4 studies)
	Pelvis rotation (right)	4.030		Ref^36^; N = 20 (20M, 0F); *x̅* = 16.0	Good (1 study)
	Pelvis anterior tilt	1.138		Ref^36^; N = 20 (20M, 0F); *x̅* = 16.0	Good (1 study)
	Peak left knee extension angular velocity	1.090		Ref^36^; N = 20 (20M, 0F); *x̅* = 16.0	Good (1 study)
	Trunk energy output: RPL to MER	1.054		Ref^24^; N = 19 (19M, 0F); *x̅*=25.1	Good (1 study)
	Lower lumber right rotation maximum	−0.864		Ref^36^; N = 20 (20M, 0F); *x̅* = 16.0	Good (1 study)
	Peak right knee extension % cocking stage	−1.163		Ref^36^; N = 20 (20M, 0F); *x̅*=16.0	Good (1 study)
	Lower trapezius strength	−1.323		Ref^47^; N = 42 (0M, 42 F); *x̅* = 45.9	Good (1 study)
	Humerothoracic angle: MER-Abduction	−1.366		Ref^4^; N = 28 (28M, 0F); *x̅* = 12.1	Good (1 study)
	Pelvis-shoulder right rotation	−2.387		Ref^36^; N = 20 (20M, 0F); *x̅* = 16.0	Good (1 study)
Acceleration phase: Acceleration	Shoulder inferior force (/BM)	1.697		Ref^49^; N = 20 (20M, 0F); *x̅* = 25.0	Good (1 study)
	Lower lumbar left rotation	1.093		Ref^36^; N = 20 (20M, 0F); *x̅* = 16.0	Good (1 study)
	Upper lumbar left lateral flexion (max)	1.064		Ref^36^; N = 20 (20M, 0F); *x̅* = 16.0	Good (1 study)
	Elbow medial force	0.943		Ref^49^; N = 20 (20M, 0F); *x̅* = 25.0	Good (1 study)
	Upper lumbar flexion (to EoR)	0.943		Ref^36^; N = 20 (20M, 0F); *x̅* = 16.0	Good (1 study)
	Pelvis posterior tilt (max)	−1.402		Ref^36^; N = 20 (20M, 0F); *x̅* = 16.0	Good (1 study)
Acceleration phase: Ball contact	Wrist extension RoM (to lower extremity injury)	0.911		Ref^20^; N = 55 (35M, 20F); *x̅*=15.4	Good (1 study)
	Humerothoracic angle: Impact – Abduction	−1.061		Ref^4^; N = 28 (28M, 0F); *x̅* = 12.1	Good (1 study)
	Anterior-Posterior ball position	−1.054		Ref^35^; N = 20 (20M, 0F); *x̅* = 16.0	Good (1 study)
	Wrist extension strength	−0.991		Ref^47^; N = 42 (0M, 42 F); *x̅* = 45.9	Good (1 study)
Follow through phase: Deceleration	Dominant shoulder tightness	2.894	CI: 0.152 to 5.636	Refs^48,59^; N = 149 (149M, 0F); *x̅* = 25.7	Limited (2 studies)
	Dominant shoulder internal rotation RoM	−1.118	CI: −2.362 to 0.126; *P* = .078; *z* = −1.760	Refs^4,5,48,50,52,58,59^; N = 424 (373M, 51 F); *x̅* = 23.0	Moderately strong (7 studies)

Abbreviations: BM, body mass; BT, ball toss; CI, confidence interval; EoR, end of range; ER, external rotation; IR: internal rotation; MEF, maximal elbow flexion; MER, maximal external rotation of the shoulder; RLP, racket low point; RoM, range of motion.

### General Risk Factors

There were 9 general risk factors that had a large or significant association with injury in tennis players (Table 4). Four of the general risk factors were from multiple studies, allowing further meta-analysis (age, body mass, height, and weekly tennis). Interestingly, the meta-analysis showed that increased age, body mass, height and weekly tennis volume all had a significant small association with increased tennis injuries, despite the low overall effect size values (*d* = 0.202; *d* = 0.209; *d* = 0.153; *d* = 0.159 for age, body mass, height and weekly tennis respectively; Tables 3 and 4). These risk factors were formed from 21, 20, 17 and 9 studies respectively, possibly explaining the significant result from meta-analysis. The 95% confidence intervals from the meta-analysis for these 4 risk factors suggested these results have a moderate strength of evidence (see Appendix 3 for forest plots).Even though the participants from all the studies forming these risk factors were from the same tennis playing populations this perhaps suggests some difficulty in precisely matching participants. Similar aged but earlier maturing players being slightly taller and heavier and playing with greater intensity, will generate greater torque/force through their joints without the resilience to injury accumulated from years of strength and conditioning, perhaps contributing to a slightly higher injury risk. This suggests care should be taken when working with age group tennis players. However, this is a small effect and perhaps due to not being able to exactly match injured and uninjured players for age, body mass and height in each study, but warrants further investigation.

Exposure to tennis has previously been suggested to be a risk factor for injuries in general.^63^ Consistent with this, despite the low overall effect size (*d* = 0.159), meta-analysis from 9 studies showed that increased weekly tennis has a significant association with increased injury (*P* = .037). Tennis players can experience considerable training loads, with professional players training 5-6 hours a day, 5-6 days a week,^7-9^ and junior players averaging up to 9 hours of tennis per week.^5^ The Acute: Chronic workload ratio (ACWR) has also been linked to injury incidence in a range of sports including tennis players.^43,44^ However, the 7 studies that looked at overall training volume showed no clear relationship with increased injury (*d* = 0.099, *P* = .245; Table 3). As well as differences in methodology, increasing numbers of certified tennis coaches^2^ may mean more structured tennis training and conditioning, allowing sustained, planned training leading to improved performance and reducing the potential for sudden increases in training load and intensity that would increase the ACWR and increase the risk of injury.^64^ In this regard, athlete monitoring, including of workloads, fatigue and athlete self-report measures of recovery, sleep, wellbeing etc. are vital to minimise the risk factors associated with tennis and other physical activity exposure.^65^ Various studies have also reported a greater risk during competition compared to practice.^66,67^ Knowledge of and applying match specific parameters (e.g., number of shots per minute, rally length, rally pace etc.^68^) to induce appropriate intensity of physiological outcomes within training should be also considered by coaches and included into the periodised plan.^69^

The racket length/weight risk factor was the only other risk factor from more than 1 study, but had a limited quality level (Table 4). This showed the perhaps unsurprising finding that longer or heavier rackets were associated with a greater risk of injury. All other general risk factors in Table 4 were from a single study. The largest of these risk factors was regularly performing a stretching routine^20^ (Figure 3, Table 4). This supports a systematic review^70^ showing static stretching does not reduce overall injury, but this review also reported preliminary evidence that stretching may reduce musculotendinous injuries. Stretching is a common activity recommended for anyone participating in an exercise programme.^71^ It is not clear what type of stretching this risk factor applies to, but the role of stretching in reducing injuries is a subject of continued research and debate.

One study in junior tennis players^20^ showed 1 of the largest risk factors for injury is previous injury, regardless of location on the body (*d* = 1.270, Figure 3, Table 4). Consistent with a number of other studies into injury risk factors in various sports, history of previous injury is a strong risk factor for subsequent injury and can become a significant barrier to maintaining a healthy active lifestyle.^66,72-75^ It is not clear why this is such a strong relationship, but this has been suggested to be due to inadequate rehabilitation or pre-existing risk factors.^20,76^

Risk factors associated with singles and doubles matchplay were explored in two studies.^20,38^ In general, doubles matchplay had little association with risk of injury. Singles matchplay (*d* = 0.444) had a higher risk of injury compared to doubles matchplay (*d* = 0.016; Figure 3). Only one study^20^ found singles matchplay to be a large risk factor for injury in junior club tennis players (*d* = 0.871), compared to a very low risk from professional women players in grand slam tournaments (*d* = 0.016^38^). This may be due to player ability and associated workload as the injured junior club tennis players had a much greater frequency of singles tennis play each week compared to the uninjured group, and may still be developing a good stroke technique.^20^ Junior players may not have been exposed to as much strength and conditioning as professional, senior players so may be more susceptible to overuse injuries.

### Preparation Phase

The preparation phase of the tennis serve is from the first sign of movement to the point of maximum shoulder external rotation.^22^ This includes the ball release, loading, and cocking stages (sometimes referred to as the drive phase^36^) and serves to store energy ready for the acceleration into ball impact in the subsequent acceleration phase.^18,22^ Key parts of the preparation phase are the loading and cocking stages, preparing to accelerate the racket into the ball at impact. A lot of focus on injuries associated with the tennis serve investigate the scapula and upper limb movements during these key phases. Coordinated movement of the humerus, scapula and clavicle are essential for normal function of the shoulder girdle and fatigue has been shown to alter scapulothoracic kinematics.^77^ However, the preparation phase includes the ball toss and any signs of scapula dyskinesis may be apparent from this early stage.

Only 4 of the 16 risk factors with a large association with injury were from more than 1 study (Table 4). During ball release, greater scapulothoracic upward rotation (*d* = 1.052) in the dominant arm had a large association with shoulder injury. A greater scapula upward rotation also shows a moderate association with injury in the cocking phase of the serve (*d* = 0.492; Figure 6). Linked to this, humerothoracic movement is important in the tennis serve preparation phase. Overhead athletes often present with excessive external rotation and limited internal rotation of the glenohumeral joint.^78^ This is consistent with tennis players often having an increased ER ROM and it has been observed that during the cocking phase, the anterior shoulder capsule may experience loads up to the equivalent of 40% of total body weight.^13,79^ Consistent with this, increased dominant arm humerothoracic external rotation showed a large association with shoulder injuries (*d* = 0.856; Table 4), and following meta-analysis greater dominant shoulder external rotation strength and ER/IR strength ratio showed a significant association with a reduction in shoulder injuries (Table 4). The wider 95% confidence intervals from the meta-analysis of the dominant shoulder ER strength and the ER/IR strength ratio suggested these risk factors had a low strength of evidence (see Appendix 3 for forest plots).

Excessive external rotation in overhead athletes has been suggested to be associated with shoulder injury.^78^ In tennis, the glenohumeral external rotation movement is largely unloaded, but excessive glenohumeral ER perhaps suggests poor scapula positioning and control for subsequent phases. Increased scapula upward rotation may be a compensation for previous injury, fatigue or lack of strength in this area. Increased scapula upward rotation, in association with diminished humeral external rotation resulting from fatigue, has been shown in arm elevation movements.^18,77^ It has even been hypothesised that decreased scapulothoracic upward rotation may increase the risk of shoulder problems.^80^ This apparent contradiction perhaps suggests that there is a goldilocks zone where the scapula rhythm is just right, with too much or too little movement being associated with injury. Range of motion is often used to assess tennis players, particularly with a view to rehabilitation following injury, to identify potential injury risk from reduced flexibility^81^ or evaluate potential for service speed (for example^82^). Shoulder IR RoM and ER RoM has also been shown to decrease and increase respectively at the end of a tennis match compared to the start.^53,83^ This has also been shown in baseball and was suggested to be due to the shortening of the posterior shoulder musculature and tightening of the posterior shoulder capsule, with skeletal adaptations also potentially contributing to the changed IR and ER motion.^84^

Muscle strength has a strong link to the risk of injury in the tennis serve (Figure 6, Table 4). Increased strength of the trapezius muscle has a moderate to large association with reduced injury risk (lower trapezius *d* = −1.323; upper trapezius *d* = −0.463). Four studies showed that an increased dominant arm ER/IR strength ratio is associated with a significant reduction in injury risk (*d* = −0.608; *P* < .001), consistent with increased dominant arm ER strength being associated with reduced injury risk (*d* = −0.384; *P* = .025; Table 4). Upper and lower body strength are clearly important in reducing injuries in the tennis serve and strength and conditioning coaches should consider the implications these results have on programme design and warm-up strategies. Indeed, increased exposure to developmentally appropriate strength and conditioning, injury in youth athletes can be reduced by as much as 50%.^65^ A reduced energy flow output from the trunk to the hand is associated with greater injury risk (*d* = −1.275; Figure 5; Table 4). This is perhaps linked to studies showing a greater knee bend maintains serve speed while reducing shoulder internal rotation torque.^17^ A previous lower limb injury was also a strong risk factor for injury risk (*d* = 1.615; Figure 5), perhaps suggesting that tennis players need to be strong enough in the upper limbs to cope with the forces being generated by the lower body and transferred up the kinetic chain, and effective whole body movement reduces injury risk.

While the largely unloaded movements in the early ball toss stage of the serve may not present a large injury risk in themselves, the altered scapula and humerothoracic movements in the subsequent phases of the serve may be a symptom of reduced leg drive and trunk rotation, leading the upper body to generate increased torque to produce greater racket and ball velocity. Indeed, high quality energy flow can result in higher ball velocity despite reduced upper limb joint kinetics.^24^ Following the ball toss stage of the tennis serve is the loading stage, when the knees bend, the trunk and pelvis rotate and the shoulders and pelvis tilt to store potential energy leading into the cocking stage, when the racket head moves down behind the body to lengthen the trajectory of the acceleration phase to follow.^22^ Many of the risk factors in this phase are from single studies, but together they can help understand the injury risks. Shoulder and pelvis lateral rear tilt leading into the cocking stage is a feature of powerful servers.^85^ A reduced pelvis and shoulder rotation has been suggested to be influenced by lower back pain, and by age and gender.^86^ Pelvis anterior tilt in the cocking stage was strongly associated with risk of LBP (*d* = 1.138; Table 4), suggesting this movement is not compatible with a powerful serve. Achieving a powerful loading stage requires optimal core stability and lateral trunk flexion throughout the range of motion.^22^ However, neither upper or lower lumbar lateral flexion have a large association with LBP in the cocking stage (*d* = −0.145 and 0.166 respectively; Figure 6).

Risk factors associated with the loading and cocking stages of the tennis serve are shown in Figures 4 and 5. A greater upward rotation of the scapula shows a moderate association with injury in the cocking stage (*d* = 0.492), consistent with the large effect from the scapula upward rotation risk factor in the ball release phase (*d* = 1.052; Table 4). A lead knee flexion greater than 15° leading to potentially greater ground reaction force (or impulse) has been recommended to reduce shoulder and elbow IR torque.^12,87^ Tennis is a sport requiring repetitive multidirectional trunk movements,^39^ but the trunk muscle profile showed that various peak torque values presented only low association with low back pain (Figure 5). However, a study into the timing of maximal angular velocities of pelvis and trunk rotations suggests later timing of these parameters in the serve action was linked to higher shoulder joint forces, lower ball velocities and greater injury risk.^49^ The greater impulse may be simply due to later timing allowing more time to generate force from the serve in the acceleration phase, or less effective coordination of upper and lower body requiring greater force generation from the upper body to achieve high serve velocity.

Campbell et al^36^ studied players with LBP and compared their serve to pain-free players. During the cocking stage in right-handed players, greater rightward lumbar rotation away from the target, larger rightward shoulder (trunk) rotation relative to the pelvis and a downwards rotation in the left hip (pelvis rotation around the sagittal axis in the fontal plane) were all associated with a reduction in LBP. An increase in pelvis rightward rotation (away from the target) however was associated with a large increased risk of LBP (*d* = 4.03; Figure 6; Table 4). The much greater pelvis right rotation in the cocking stage of players with a history of back pain injury perhaps suggests they are “winding up” for a high velocity serve, thus creating greater loads at the distal joints. The increased injury risk with greater pelvis right rotation is the opposite of lower lumbar right rotation (*d* = −0.864), showing that pelvis rotation is very different to lower lumbar rotation. The greater lumbar rotation and reduced pelvic rotation in pain-free players suggests these players are effectively storing potential energy ready to be utilised through the stretch-shortening cycle (SSC) action, and trunk and lumbar mobility is needed to reduce the risk of LBP. Similarly, the stability of the pelvic girdle is important to reduce LBP in the tennis serve. Greater pelvis anterior tilt (*d* = 1.138) and greater right lateral pelvic tilt (*d* = 0.743) is associated with increased risk of LBP.^36^

The timing of these movements may be crucial for an effective tennis serve. If the lumbar spine has greater rotation relative to the pelvis, the impact of the stretch-shortening cycle will enhance the force generated in the acceleration phase of the serve and produce greater ball velocity.^88^ Misalignment or later muscle firing of the pelvis towards the target suggests an ineffective separation between pelvis and lumbar trunk to generate a stretch shortening cycle and transfer energy through proximal to distal sequencing. The reduced stretch-shortening cycle (and subsequent recoil) contribution will reduce the torque transferred to the ball impact and will require greater muscle activation in distal segments to “catch up” or make good this shortfall. Many of the risk factors with a large effect size in the preparation phase are each from a single study, but the coordinated movement they suggest to reduce injury risk in the tennis serve is worth further investigation.

### Acceleration Phase

The acceleration phase follows the loading and cocking/drive stages and begins at the point of maximal external rotation of the serving arm shoulder as it starts to internally rotate, and ends with ball impact.^22^ It has been the subject of several investigations covering specific anatomical locations. Campbell et al^35^ looked at the lumbar spine as a whole and Campbell et al^36^ separated the lumbar spine into upper and lower regions. Gillet et al^4^ looked at shoulder movements and Martin et al^24^ looked at energy flow. The phase has also been identified with different names including the forward swing phase (or from the racket low point to ball impact),^35,36^ the late cocking phase and acceleration phase (racket low point to maximal external rotation to ball impact).^24^ This present study will use the term acceleration phase for the period from racket low point to ball impact.

This phase can happen very fast - elite tennis players can move the serving arm from maximal glenohumeral joint external rotation to ball impact in 0.01 second.^89^ The high level of muscle contraction needed to achieve these fast movements can predispose players to injury, particularly if combined with poor technique.^22,23,81^ All the risk factors aligned to the acceleration phase are each from a single study. All the studies had a “Good” quality level from the Downs and Black analysis but can’t be compared to risk factors from multiple studies. Greater shoulder inferior force (downward movement of humeral head), elbow medial force, shoulder anterior force (forward movement of humeral head) and shoulder horizontal abduction torque during the serve action all have moderate to high associations with injury risk (Table 4; Figure 7). However upper body strength has an important role in the tennis serve. The pectoralis major, subscapularis, latissimus dorsi, serratus anterior, deltoid, trapezius and triceps are all highly active during humerus IR in the tennis serve acceleration phase, often above 100% of maximum voluntary isometric contraction.^25^

In the acceleration phase, greater lower lumbar left rotation (towards the target in a right-handed player; *d* = 1.093) and upper lumbar left lateral flexion (*d* = 1.064) have large associations with injury (Table 4). Pain-free players showed little lumber rotation or pelvis rotation towards the target, and less upper lumbar left lateral flexion, but greater leftward shoulder rotation relative to the pelvis and larger pelvic posterior tilt.^36^ Indeed, previous research has reported excessive anterior pelvic tilt can cause femoroacetabular impingement and sacroiliac joint pain.^90^ This suggests the players are realising the benefits of storing potential energy during the previous cocking stage, and using a solid trunk unit and movements of the lumbar and pelvis regions to realise the SSC and increase the ball velocity at impact. The kinetic chain in the tennis serve allows the transfer and build-up of potential energy through a proximal to distal sequence starting from the ground and lower limbs, through to the racket. It has been suggested that the major contributors to the racket head velocity in the tennis serve are the upper arm rotation and wrist flexion.^61^ However, for this to work effectively the legs and trunk provide the proximal base to generate initial forces leading to the maximisation of ball velocity at the distal end of the sequence. This would suggest that strong trunk musculature is important for the acceptance of forces from the lower body, and subsequent transfer to the upper limb and racket, in an effective and efficient tennis serve. Mathematical modelling of the kinetic chain has shown that reducing kinetic energy from the trunk by 20% requires an increase of 34% in hand velocity to maintain the kinetic energy at the hand.^22^

The literature suggests that trunk strength and stability may play a large part in control of the tennis serve. Increased pelvis rotation shows a moderate association with increased injury in the acceleration phase (*d* = 0.644; a lot lower than in the cocking stage when increased pelvis rotation is associated with a large risk of LBP injury: *d* = 4.030; Figures 6 and 7 respectively). This difference between the cocking and acceleration stage is also seen with the pelvis-shoulder rotation, but greater shoulder rotation relative to the pelvis is associated with a moderate reduction in risk of injury in the acceleration stage (*d* = −0.656; Figure 7) and a large reduction in injury risk in the cocking stage (*d* = −2.387; Table 4). This suggests that when players are able to separate upper and lower body (through trunk rotation and control/stability through pelvis rotation) there is a lower associated risk of injury and perhaps a greater ability to generate stretch-shortening cycle benefits to transfer forces through to the upper body without imposing excessive force on the shoulder joints. Martin et al^49^ showed the importance of the trunk contribution to reduce upper limb joint kinetics while maximising ball velocity. Greater utilisation of the trunk angular momentum increases the contraction of the shoulder internal rotators and thereby the ball velocity. Trunk strength has been shown to be an important part of the kinetic chain for performance and injury prevention in other sports, such as swimming^91^ and baseball pitching.^92^ Effectively linking the upper and lower body through strengthened trunk musculature during the activity will promote increased service velocity and reduced injury risk. This can be reflected in the efficiency of the serve’s kinetic chain energy flow and the role of the pelvis and spine mechanics in the serve. A much greater quality of energy flow from the trunk to the hand + racket segment was seen in uninjured players compared to injured players.^24^ Greater energy output from the trunk in the cocking phase and the acceleration phase has a much higher association with injury (*d* = 1.054 and 0.642 respectively), despite similar energy inputs to the hand and racket (*d* = 0.135 and −0.052 respectively), showing less effective energy transfer from proximal to distal segments in the injured players (Table 4; Figures 6 and 7). With reduced energy transfer from the trunk to the arm, it is possible that the shoulder and arm must generate increased torque in order to maintain these similar energy outputs.

The lumbar rotation movement in the cocking and acceleration stages of the tennis serve had interesting associations with back pain.^36^ Greater lower lumbar left rotation (towards the target) in the acceleration stage is associated with higher risk of LBP (*d* = 1.093; Table 4). However, greater lower lumbar right rotation (away from the target) in the cocking stage is associated with a reduction in injury risk (*d* = −0.864; Table 4). This earlier lower lumbar rotation movement, synchronised with the pelvis rotation, in the cocking stage of uninjured players is consistent with building the potential energy for the serve in the cocking stage, then stabilising the pelvis followed by the trunk (through sequential deceleration of the segments) in the acceleration stage to allow effective transfer of the energy into the ball at impact.

#### Ball Contact

Ball impact is the culmination of the proximal to distal sequencing that takes place from initiation of movement in the tennis serve. The position of the ball will be partly determined by the ball toss, but the position of the ball on the racket at the point of contact is also important. Campbell et al ^35^ found that making impact with the ball in a slightly more anterior (*d* = −1.054; Table 4) position was associated with lower risk of back pain, and impact in a more superior position (*d* = 0.745; i.e., increased vertical height) was associated with greater risk of back pain (Figure 8). A more inferior and anterior position at impact may mean (technique dependant) the shoulder is in less external rotation with less glenohumeral flexion at ball impact, reducing the torque on the scapulothoracic joint and less force in the sub-acromial space, so reducing the impact on the shoulder structures. However, Gillet et al^4^ found a greater humerothoracic ER angle (*d* = −0.542) and greater humerothoracic abduction angle at ball impact (*d* = −1.061) were both associated with a reduced risk of injury in the tennis serve. A greater humerothoracic ER angle at ball impact suggests a more posterior ball impact and a greater humerothoracic abduction angle suggests a more superior ball impact (serving arm pointing more upwards^93^). This contradiction may be down to differences in method used and injury label. Participants in the Campbell et al study^35^ served within 5 km/hour of their maximum serve speed and recorded incidence of lower back pain; the Gillet et al study^4^ used a slow serve motion, 5-times slower than a normal serve, and used history of shoulder problems.

Analysis of arm and shoulder movements at ball impact during slow velocity tennis serves also revealed that a greater scapulothoracic internal and upward rotation at ball impact had a moderate association with injury (*d* = 0.505 and 0.492 respectively). This may be a consequence of the different serve speeds. As discussed earlier, the increased scapula movement may also be compensating for reduced glenohumeral elevation consequent to injury. This suggests that players with reduced scapula control and stabilisation when producing greater humerothoracic movement in the serve have an increased risk of shoulder injury. From an applied perspective, practitioners should be encouraged to assess scapula stability and to provide interventions where required to reduce this risk.

The wrist plays a key part in the tennis serve, reaching peak velocity approximately 0.03 second before ball impact^94^ but after maximal shoulder external rotation is achieved (at approximately 0.09 second before impact^89^). Greater wrist flexion and extension strength were associated with less risk of injury in recreational tennis players (*d* = −0.757 and −0.991 respectively; Figure 8; Table 4).Upper arm internal rotation determines 54% of the racket head velocity, but wrist flexion adds 31% to the final racket head velocity.^61^ Effective use of the wrist just prior to and at the point of impact will help effectively transfer the momentum and potential energy stored during the drive phases of the serve. However, advances in racket technology and serve speeds since 1995 may have had an impact here.

### Follow Through Phase: Deceleration and Finish Stages

The deceleration stage in the tennis serve requires eccentric muscle contraction in both the upper and lower body in response to the previous cocking and acceleration stages. Similar to the follow-through phase of baseball pitching^95^ the deceleration stage of the tennis serve can present considerable load on the controlling musculature of the shoulder, elbow and wrist following the acceleration phase. The posterior rotator cuff muscles (supraspinatus and infraspinatus) have an average activation level of 30% to 35% of maximum as the humerus is decelerated during this stage.^25^ A strong link to shoulder pain in this stage is the dominant shoulder tightness and the dominant shoulder IR RoM (Figure 9). These 2 risk factors are both from multiple studies that have a limited to moderately strong evidence level (Table 4) but the 95% confidence intervals from the meta-analysis of the dominant shoulder IR RoM suggest a low strength of evidence for this risk factor (see Appendix 3 for forest plots). The 2 movements are related as a tighter posterior shoulder will limit the internal rotation range of motion, thus increasing the load on the shoulder during the deceleration stage. The muscles involved in the serve action all have moderate to high activity during the follow-through phase of the serve,^25^ so may all contribute to risk of injury. Shoulder IR RoM has been shown to reduce with the fatigue of a tennis match.^83^ Our review suggests a reduction in dominant shoulder IR RoM (*d* = −1.118; *P* = .078; Table 4; Figure 9) has a larger association with shoulder injury than GIRD (*d* = 0.748; Figure 7), consistent with suggestions that limited IR RoM is more associated with shoulder problems than GIRD in the tennis serve, despite GIRD being linked with rotator cuff impingements and labrum tears.^50^

Players with a previous lower limb injury may also have a higher risk of injury in this phase (*d* = 1.615; Figure 5) especially with eccentric muscle action taking place here (a factor known to induce greater muscle damage and generate higher forces than concentric/isometric contraction^96^). The mobility and conditioning of the lower limbs can also be linked to injury risk in the finish phase, when landing from the serve and moving into a subsequent shot or movement across the court, as deficits in hip rotation RoM have been linked to higher lumbar spine mechanical stress in tennis players.^53^ Greater non-dominant and dominant leg hip extension RoM have moderate associations with reduced risk of LBP, perhaps contribute to a reduction in lumbar spine mechanical stress (Figure 10).

#### Limitations

All the studies included in the current review used a variety of methodological characteristics and statistical analysis to collect, analyse and report data and findings. The average age of participants in the studies included in this review was 23.2 years (SD = 9.8; range: 9-75 years), so the risk factors associated with the specified stages of the serve appears to be most representative for these populations and may not be generalisable to other groups. Differences in the use of terminology between professions can also cause confusion. We have tried to be consistent in our use of terminology in the text. Many of the risk factors in this review were from only 1 study. This can limit the comparison when comparing to the 16 risk factors that were made up of 3 or more studies.

There are also number of strengths we would like to highlight:

We included all suitable studies that met the inclusion criteria, so we used a number of methods to convert the results to Cohen’s *d* effect size. This assumed the studies were all comparable, but they all used different methodological approaches. We took the opinion that even if these assumptions do not hold exactly, omission of any studies using different forms of effect size would result in a potential loss of information and reduce the depth of the review.^28^

PRISMA guidelines were followed, and stringent inclusion and exclusion criteria were adhered to in the selection of the review articles ensuring transparency and robustness throughout. Downs and Black checklist scores were calculated for all studies. Where more than 10 studies formed a risk factor, there was no evidence of publication bias from funnel plots.

The review used broad inclusion criteria for the paper type (e.g., primary studies, conference presentations, Doctoral theses) resulting in an extensive literature search, enabling literature to be included that would otherwise be missed.

Two articles were translated from different languages (German and Portuguese) as it was deemed important to include these studies as they provided information in line with the research questions of this paper.

## Conclusion

This study identified 130 injury risk factors associated with injury in the tennis serve. Of these risk factors, 36 were considered large (*d* ⩾ 0.8 or *d* ⩽ 0.8), and 7 were significant following meta-analysis. Only 10 of the 36 risk factors with a large effect size were from 2 or more studies, with the rest being from single studies. From the meta-analysis, there is moderate strength of evidence that an increase in age, height, body mass and weekly tennis volume are associated with an increase in risk of injury in tennis players aged 11 to 46 years (mean age: 21 years). This perhaps suggests earlier maturing players, being slightly taller, heavier and playing with greater intensity may be at a higher risk of injury during the serve. Wider confidence intervals suggest there is lower strength of evidence that dominant shoulder external rotation strength, ER/IR strength ratio and dominant shoulder internal rotation RoM are associated with reduced risk of injury in tennis players aged 11 to 46 years (mean age: 30 years). This highlights the importance of coach and player awareness of these factors in tennis practice and strength and conditioning programmes. Furthermore, evidence-based continuous professional development and coach education materials could be developed using the results of this paper.

The diverse range of participants included in this review displayed some heterogeneity in the data presented, definition of injury and participant numbers. The average Downs and Black checklist score for the risk factors considered large ranged from fair to good. The 4 risk factors calculated from more than 10 studies showed no evidence of publication bias from funnel plots. Further studies investigating injured and uninjured tennis players using standardised research methodology to allow comparison to previous work would strengthen the evidence base and further clarify the important risk factors associated with injury in the tennis serve.

## Supplemental Material

sj-docx-1-rpo-10.1177_27536351251374616 – Supplemental material for Injury Risk Factors of the Tennis Serve: A Systematic Review and Meta-Analysis Supplemental material, sj-docx-1-rpo-10.1177_27536351251374616 for Injury Risk Factors of the Tennis Serve: A Systematic Review and Meta-Analysis by John Bradley, Ben L. Langdown, David Bowmaker and Stewart Kerr in Advances in Rehabilitation Science and Practice

## Figures and Tables

**Figure:**
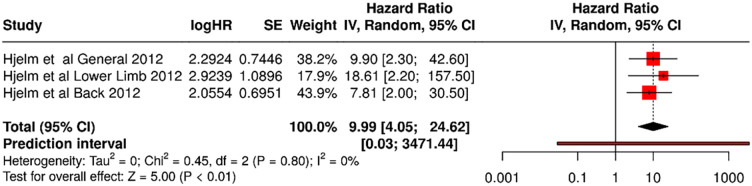
A: Previous injury risk factor.

**Figure:**
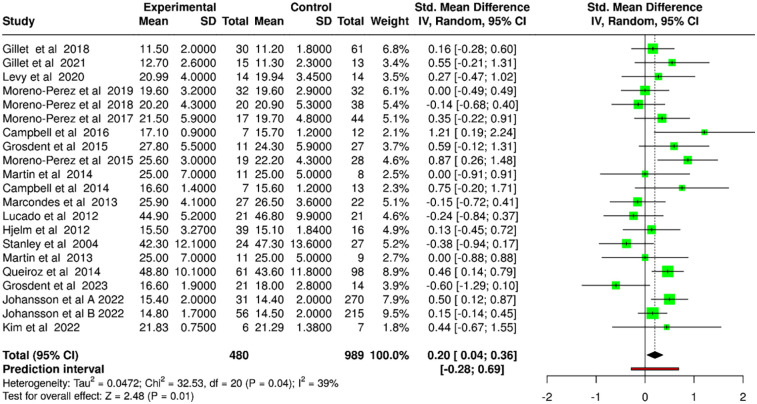
B: Age risk factor.

**Figure:**
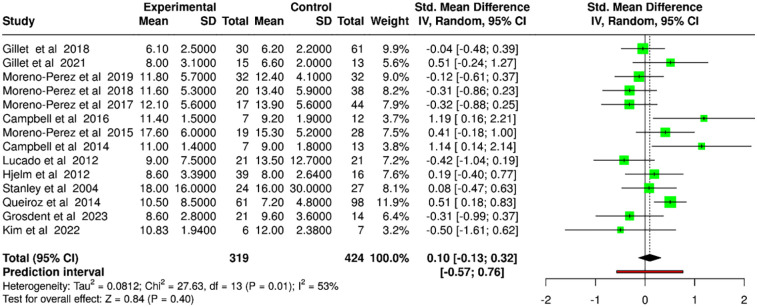
C: Years playing tennis risk factor.

**Figure:**
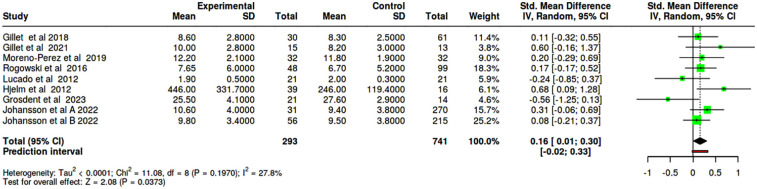
D: Weekly tennis risk factor.

**Figure:**
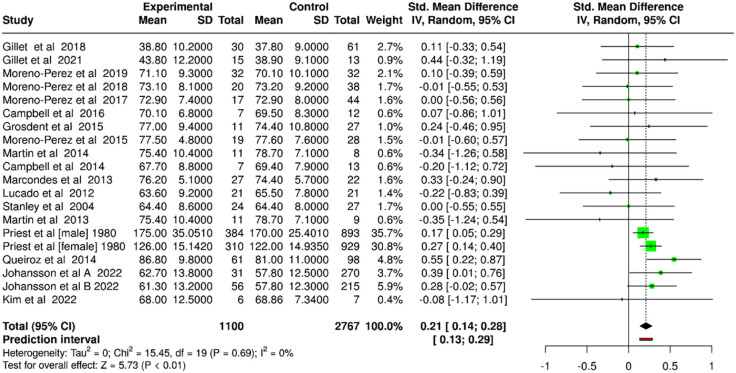
E: Body mass risk factor.

**Figure:**
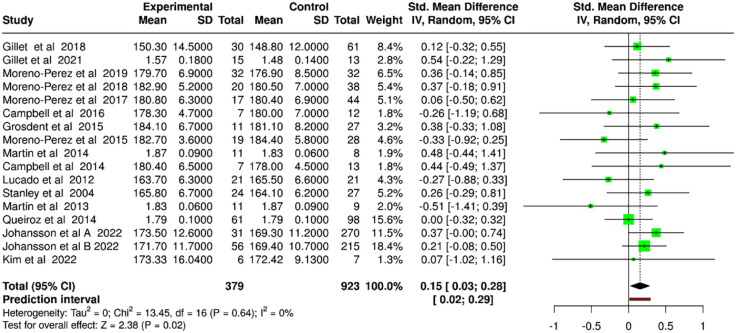
F: Height risk factor.

**Figure:**
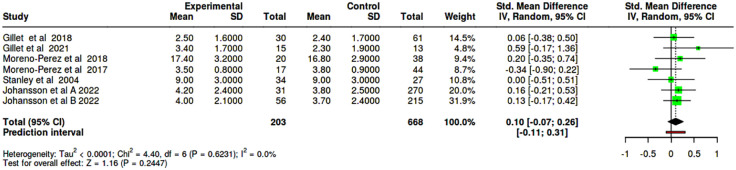
G: Training volume risk factor.

**Figure:**
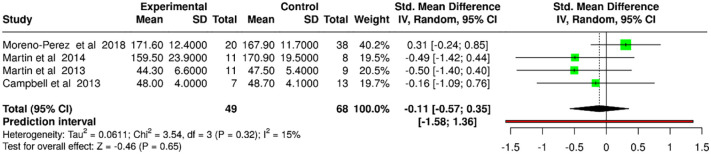
H: Serve speed/racket velocity risk factor.

**Figure:**
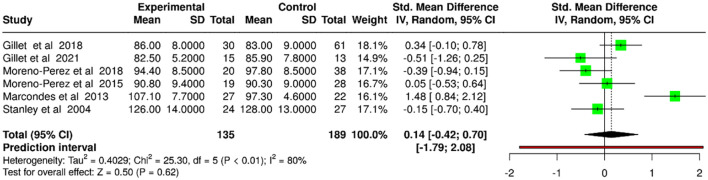
I: Dominant shoulder External Rotation Range of Motion risk factor.

**Figure:**
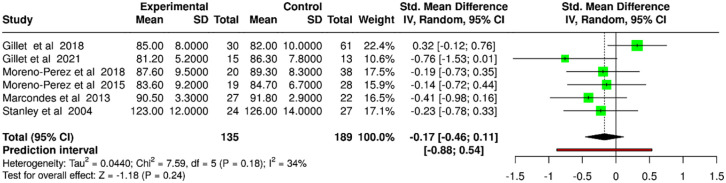
J: Non-dominant shoulder External Rotation Range of Motion risk factor.

**Figure:**
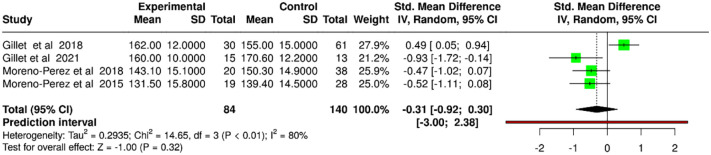
K: Dominant shoulder Total Arc of Motion risk factor.

**Figure:**
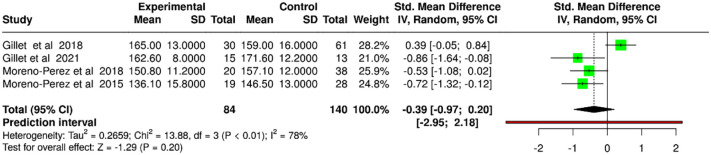
L: Non-dominant shoulder Total Arc of Motion risk factor.

**Figure:**
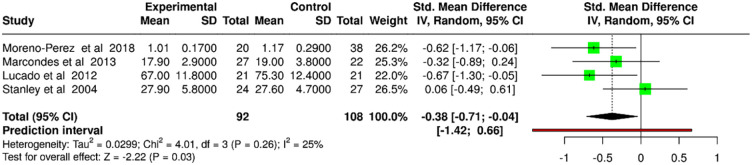
M: Dominant shoulder External Rotation strength risk factor.

**Figure:**
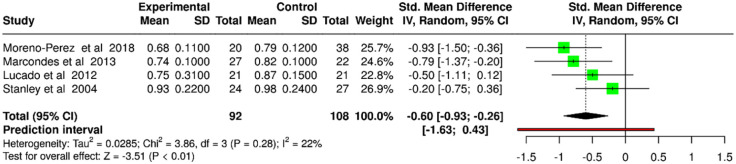
N: Dominant shoulder External Rotation/Internal Rotation strength ratio.

**Figure:**
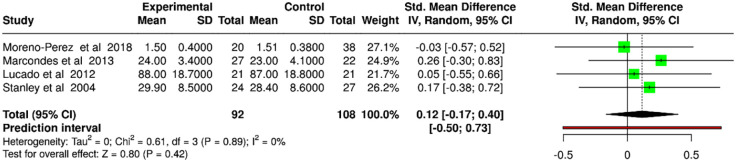
O: Dominant shoulder Internal Rotation strength risk factor.

**Figure:**
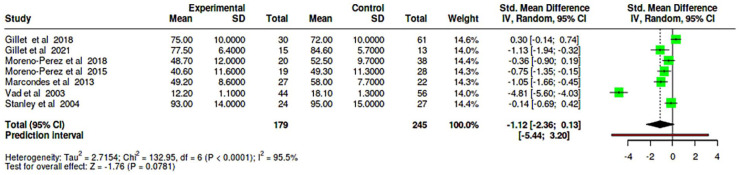
P: Dominant shoulder Internal Rotation Range of Motion risk factor.

## Appendix 1. List of the 8 Databases Searched in This Study, the Initial Results and an Example (PubMed) of the Search Strategy Used

**Table:**

Database	Results	Example search strategy (PubMed)
PubMed (Open Access)	1627	(“Tennis”[Title/Abstract] AND “risk factor*”[Title/Abstract] AND “injur*”[Title/Abstract] AND “Pain”[Title/Abstract]) OR (“Tennis”[Title/Abstract] AND “risk factor*”[Title/Abstract]) OR (“Tennis”[Title/Abstract] AND “injur*”[Title/Abstract]) OR (“Tennis”[Title/Abstract] AND “Pain”[Title/Abstract]) OR (“tennis/injuries”[MeSH Terms] AND “risk factors”[MeSH Terms]) OR (“tennis/physiology”[MeSH Terms] AND “risk factors”[MeSH Terms]) OR (“Tennis”[Title/Abstract] AND “athletic injuries”[MeSH Terms]) OR (“Tennis”[Title/Abstract] AND “risk factors”[MeSH Terms]) OR (“tennis/injuries”[MeSH Terms] AND “prospective studies”[MeSH Terms]) OR (“tennis/injuries”[MeSH Terms] AND “retrospective studies”[MeSH Terms])
Medline (EBSCOhost)	1497	
SPORTDiscus (EBSCOhost)	1631	
Academic Search Complete (EBSCOhost)	1503	
AMED (EBSCOhost)	195	
CINAHL (EBSCOhost)	784	
Web of Science (Clarivate Analytics)	1285	
ScienceDirect (Open Access)	442	
Total	8964	
Total after removal of duplicates	4337	

## Appendices

### Appendix 2: Results of Methodological Quality Assessment for Included Articles Based on the Downs and Black and Blacks Checklist^33^ Adapted for This Study

*Downs and Black*^33^ *checklist heading number* 1: Is the hypothesis/aim/objective of the study clearly described?; 2: Are the main outcomes to be measured clearly described in the Introduction or Methods section?; 3: Are the characteristics of the patients clearly described?; 6: Are the main findings of the study clearly described? 7: Does the study provide estimates of the random variability in the data for the main outcomes? 10: Have the actual probability values been reported for the main outcomes, except where the probability values is less than 0.001?; 11: Were the participants asked to participate in the study representative of the entire population from which they were recruited?; 12: Were those participants who were prepared to participate representative of the entire population from which they were recruited?; 16: If any of the results of the study were based on “data dredging,” was this made clear?; 18: Were the statistical tests used to assess the main outcomes appropriate?; 20: Were the main outcome measures used accurate (valid and reliable)?; 21: Were the patients in different intervention groups (trials and cohort studies) or were the cases and controls (case-control studies) recruited from the same population?; 22: Were study participants in different intervention groups (trials and cohort studies) or were the cases and controls (case-control studies) recruited over the same period of time?; 25: Was there adequate adjustment for confounding in the analyses from which the main findings were drawn?; 27: Did the study have sufficient power to detect a clinically important effect where the probability value for a difference being due to change is less than 5%?.

**Table:**

Study	Downs and Black (1998) checklist question number															Total	Quality Level
	Reporting						External validity		Internal validity: bias			Internal validity: confounding			Power analysis	Out of 15	
	1	2	3	6	7	10	11	12	16	18	20	21	22	25	27		
Campbell et al^35^	1	1	1	1	1	1	1	1	1	1	1	1	0	1	1	**14**	**Good**
Campbell et al^36^	1	1	1	1	1	1	0	1	1	1	1	1	0	1	1	**13**	**Good**
Campbell et al^37^	1	1	1	1	1	1	0	0	1	1	1	1	0	1	0	**11**	**Good**
Dakic et al^38^	1	1	1	1	1	1	1	1	1	1	1	1	1	1	0	**14**	**Good**
Gillet et al^5^	1	1	1	1	1	1	0	0	1	1	1	1	0	1	0	**11**	**Good**
Gillet et al^4^	1	1	1	1	1	1	0	0	1	1	1	1	0	1	0	**11**	**Good**
Grosdent et al^39^	1	1	1	1	1	1	1	1	1	1	1	1	0	1	0	**13**	**Good**
Dakic et al^38^	1	1	1	1	1	1	0	0	1	1	1	1	1	1	1	**13**	**Good**
Hang and Peng^41^	1	1	1	1	0	0	0	0	1	1	0	1	1	1	0	**9**	**Fair**
Hjelm et al^20^	1	1	1	1	1	1	1	1	1	1	1	1	1	1	0	**14**	**Good**
Jörger and Leonhard^42^	1	1	1	1	1	0	0	0	1	1	0	1	1	1	0	**10**	**Fair**
Johansson et al^44^	1	1	1	1	1	1	0	1	1	1	1	1	1	1	0	**13**	**Good**
Johansson et al^43^	1	1	1	1	1	1	0	1	1	1	1	1	1	1	0	**13**	**Good**
Kim et al^45^	1	1	1	1	1	1	0	0	1	1	1	1	0	1	0	**11**	**Good**
Levy et al^46^	1	1	1	1	1	1	0	0	1	1	1	1	1	1	0	**12**	**Good**
Lucado et al^47^	1	1	1	1	1	1	0	0	1	1	1	1	0	1	1	**12**	**Good**
Marcondes et al^48^	1	1	1	1	1	0	0	0	1	1	1	1	1	1	0	**11**	**Good**
Martin et al^49^	1	1	1	1	1	1	0	0	1	1	1	1	0	1	0	**11**	**Good**
Martin et al^24^	1	1	1	1	1	1	0	0	1	1	1	1	0	1	0	**11**	**Good**
Moreno-Pérez et al^50^	1	1	1	1	1	1	1	1	1	1	1	1	1	1	0	**14**	**Good**
Moreno-Pérez et al^51^	1	1	1	1	1	1	0	1	1	1	1	1	1	1	0	**13**	**Good**
Moreno-Pérez et al^52^	1	1	1	1	1	1	0	1	1	1	1	1	1	1	0	**13**	**Good**
Moreno-Pérez et al^53^	1	1	1	1	1	0	0	1	1	1	1	1	1	1	0	**12**	**Good**
Nigg et al^54^	1	1	1	1	1	0	0	1	1	1	1	1	1	1	0	**12**	**Good**
Priest et al^55^	0	1	1	1	0	0	0	1	1	1	1	1	1	1	0	**10**	**Fair**
Queiroz et al^56^	1	1	1	1	1	1	0	0	1	1	1	1	0	0	0	**10**	**Fair**
Rogowski et al^57^	1	1	1	1	1	0	0	1	1	1	1	1	0	1	0	**11**	**Good**
Stanley et al^58^	1	1	1	1	1	1	0	1	1	1	1	1	0	1	1	**13**	**Good**
Vad et al^59^	1	1	1	1	1	0	0	0	1	1	1	1	0	1	0	**10**	**Fair**
